# Hesperidin Alleviates Methotrexate-Induced Memory Deficits via Hippocampal Neurogenesis in Adult Rats

**DOI:** 10.3390/nu11040936

**Published:** 2019-04-25

**Authors:** Salinee Naewla, Apiwat Sirichoat, Wanassanan Pannangrong, Pornthip Chaisawang, Peter Wigmore, Jariya Umka Welbat

**Affiliations:** 1 Department of Anatomy, Faculty of Medicine, Khon Kaen University, Khon Kaen 40002, Thailand; salinee_rtu@yahoo.com (S.N.); apiwsi@kku.ac.th (A.S.); wankun@kku.ac.th (W.P.); 2 Faculty of Medical Science, Nakhonratchasima College, Nakhon Ratchasima 30000, Thailand; pornthip.CSW@gmail.com; 3 School of Life Sciences, Medical School, Queen’s Medical Centre, Nottingham University, Nottingham NG7 2RD, UK; peter.wigmore@nottingham.ac.uk; 4 Neuroscience Research and Development Group, Khon Kaen University, Khon Kaen 40002, Thailand

**Keywords:** methotrexate, hesperidin, memory, neurogenesis

## Abstract

Methotrexate (MTX), a folic acid antagonist, is widely used in cancer treatment. However, treatment with MTX reduces hippocampal neurogenesis, leading to memory deficits. Hesperidin (Hsd) is a flavonoid glycoside that promotes anti-inflammation, acts as an antioxidant, and has neuroprotective properties. Consumption of Hsd enhances learning and memory. In the present study, we investigated the protective effects of Hsd against MTX-induced impairments of memory and neurogenesis; male Sprague Dawley rats were administered with a single dose of MTX (75 mg/kg) by intravenous (i.v.) injection on days 8 and 15 or Hsd (100 mg/kg) by oral gavage for 21 days. Memory was tested using novel object location (NOL) and novel object recognition (NOR) tasks. Immunofluorescence staining of Ki-67, bromodeoxyuridine (BrdU), and doublecortin (DCX) was performed to assess cell proliferation, survival, and immature neurons. The data showed that Hsd and MTX did not disable locomotor ability. The MTX animals exhibited memory deficits in both memory tests. There were significant decreases in the numbers of cell proliferation, survival, and immature neurons in the MTX animals. However, co-administration with MTX and Hsd alleviated memory loss and neurogenesis decline. These results revealed that Hsd could protect against MTX side effects in the animals in this study.

## 1. Introduction

Neurogenesis is a process of new neural generation from neural stem cells (NSCs)/neural progenitor cells (NPCs) in the brain. It occurs during embryogenesis, in the early postnatal stages, and throughout life in the adult mammalian brain [1,2,3,4]. The subventricular zone (SVZ) of the lateral ventricle and the subgranular zone (SGZ) of the dentate gyrus (DG) in the hippocampus are two specific areas where neurogenesis develops. Neurogenesis in the SGZ is mainly associated with memory functions [5]. There are various factors that interfere with neurogenesis such as aging, physiology, and environment. Several studies have demonstrated that certain medications can induce memory impairment [6,7,8], which can cause an inability to acquire new memories, lack of recall, decreased attention, and increased confusion [9,10,11,12].

Methotrexate (MTX) is a drug used in the treatment of several cancers. It is an analog of folic acid that inhibits the enzyme dihydrofolate reductase (DHFR) [13]. This effect results in the inhibition of DNA and RNA synthesis in the S-phase of the cell cycle [14]. MTX leads to toxicity in neural stem cells, including significant declines in cell division and increases apoptosis within the SGZ of the DG [15,16]. Recently, several reports have shown that MTX causes alterations to memory ability and interferes with the neurogenesis process in terms of both physiology and pathology, which may lead to a breakdown of the memory system [17,18,19]. One previous animal study used a novel location recognition test to show that MTX treatment (75 mg/kg) causes spatial memory impairment using novel location recognition test [6]. MTX has also been shown to reduce cell proliferation and survival based on Ki-67 and bromodeoxyuridine (BrdU) expression in the DG. Apoptosis of NSCs and reductions in the number of doublecortin (DCX)-positive cells in the hippocampal DG of the adult mouse after MTX treatment have also been reported [15,17,20]. In addition, MTX generates oxidative stress by up-regulating reactive oxygen species (ROS) formation, which initiates cellular damage within the CNS [21,22,23].

Hesperidin (Hsd), a flavanone glycoside found in citrus fruits (such as oranges and lemons), some vegetables, green tea, and wine [24], has been reported to exhibit anti-inflammatory, antioxidant, antiviral, antibacterial, and anti-apoptotic efficacy [25,26]. Recent studies have revealed that Hsd improves memory and increases antioxidant activity, which is effective at ameliorating the deficits caused by various neurodegenerative diseases [26,27,28,29,30]. In addition, Hsd increases neuronal cell pro-survival pathway activity (extracellular signal-regulated protein kinases 1 and 2 [ERK1/2], which leads to the expression of proteins implicated in memory formation [31,32]. Wang et al. (2014) reported that Hsd (100 mg/kg/day) enhances memory and antioxidant activity [33]. Another study reported that Hsd has the potential to modulate brain functions, such as synaptic plasticity, which are related to learning and memory [34]. It has been reported that Hsd (200 mg/kg) administration for five days restores decreases of Ki-67-positive cells caused by methotrexate injection in small intestine [35].

Therefore, this study is designed to investigate the efficacy of Hsd in protecting against MTX-induced memory impairment and neurogenesis reduction in the SGZ in the hippocampus. Impaired memory was measured using the novel object location (NOL) and novel object recognition (NOR) tasks, whereas neurogenesis was quantified by Ki-67, BrdU, and DCX staining.

## 2. Materials and Methods

### 2.1. Animals and Drug Administration

Five-week-old male Spraque-Dawley animals (weight: 180–220 g) were purchased from Nomura Siam International Co., Ltd. (Bangkok, Thailand). Animals were housed four to a cage and maintained under standard laboratory conditions consisting of a 12-h light/12-h dark cycle with controlled temperature (25–28 °C). The animals were able to access food and water ad libitum. The protocol for this study was affirmed by Animal Ethics Committee of Khon Kaen University (Khon Kaen, Thailand; permit number ACUC-KKU-51/60 and ACUC-KKU-28/61). 

Animals were randomly assigned into one of four groups (*n* = 12 animal/group). In the vehicle group, animals received 1 mL/kg of saline solution (Nacl 0.9%) by intravenous (i.v) injection to the tail vein on days 8 and 15 and propylene glycol (100 mL/kg/day, Ajax Finechem Pty Ltd., Auckland, New Zealand) by oral gavage daily throughout the 21 days of the treatment. Animals in the hesperidin (Hsd) group received Hsd (100 mg/kg, ChemFaces Biochemical, Wuhan, China) by oral gavage for 21 days. Animals in the methotrexate (MTX) group were intravenously injected with MTX (75 mg/kg, pharmachemie BV, Haarlem, Netherlands) on days 8 and 15. In the methotrexate plus hesperidin (MTX + Hsd) group, animals were administered with MTX (on days 8 and 15 by i.v. injection) and Hsd (for 21 days by oral gavage) at the same doses as in the MTX and Hsd groups. After MTX injection, leucovorin (LCV) was intraperitoneally injected to the animals at a dose of 6 mg/kg after 18 hours and at a dose of 3 mg/kg after 26, 42, and 50 hours. The administration was similar to the application of patient treatment. LCV is used in clinical practice to mitigate the cytotoxicity of MTX [6].

Intraperitoneal injection of Bromodeoxyuridine (BrdU; 100 mg/kg, Sigma Aldrich, Saint Louis, MO, USA) was given to all groups on days 6, 7, and 8 at a volume of 5 mL/kg.

### 2.2. Behavioral Testing

Novel object location (NOL) and novel object recognition (NOR) tests were used to determine memory function after the drug administration. The tests were adapted from previous studies [8,36,37] and recorded using a video tracking system (EthoVision^®^, XT Version 12, Noldus, Wageningen, Netherlands). One day before NOL and NOR testing, each animal was habituated to the environment of the arena (open field black acrylic 50 cm × 50 cm × 50 cm) without objects for 30 min. The behavioral tests were carried out in familiarization and choice trials. The arena and objects were cleaned with 20% ethanol between each trial.

In the familiarization trial for the NOL test, rats explored two similar objects put in different positions (locations 1 and 2) for 3 min. The animals were then moved from the area to their cages for 15 min. In the choice trial, the animals were enabled to explore two similar objects, one of which was placed in the same location as before (familiar location; FL) and the other of which was placed in a new location (novel location; NL), for 3 min. For the NOR test, the animal was placed into the center of the arena to explore two similar objects placed in disparate locations (objects 1 and 2) for 3 min. The animals were subsequently returned to their home cages for 15 min. During the choice trial, the animals were put back to the arena and enabled to explore one familiar object (FO) and a novel object (NO) for 3 min.

Object exploration time in both tests was scored during the time the animals were exploring the objects, which was characterized by touching or sniffing within 2 cm of the objects [36]. The exploration time for each object was used to calculate and modify to preference index (PI). The PI was described as the length of time that the animals explored novel object or novel location divided by the time of total exploration and then multiplied by 100. In order to examine intact memory, the PI was analyzed in comparison to 50% chance [38,39].

### 2.3. Tissue Preparation

After the memory tests, all animals were killed by very fast stunning and cervical dislocation. The brains were rapidly taken out and then divided along the median plane, and then preserved in 30% sucrose solution for 3 h at 4 °C. Next, each hemisphere of the brain was embedded in OCT-compound (Thermo fisher scientific, Karlsruhe, Germany), frozen quickly in liquid nitrogen-cooled isopentane, and collected at −80 °C for immunofluorescence.

### 2.4. Immunofluorescence

Cell proliferation in the DG of the hippocampus was determined using Ki-67 staining. Sections were cut along the frontal plane at 20 µm thickness from the Bregma point, −2.3 to −6.3 mm, to obtain the whole DG using a cryostat (Cryostat Series HM 550 Microm international; Walldorf, Germany), and every 15th section was selected (9 sections per brain) [8,40]. Ki-67 staining was carried out as earlier reported [37,39,40]. In brief, sections were preserved with 0.5% paraformaldehyde (PFA; Sigma-Aldrich, Inc., Saint Louis, MO, USA) (pH 7.4) for 3 min and then incubated with mouse monoclonal Ki-67 primary antibody (1: 150, Vector Laboratory, Inc., Burlingame, CA, USA) for 1 h at room temperature. After washing with phosphate buffer saline (PBS), sections were incubated with Alexa Fluor 488 secondary antibody (1:300, Invitrogen, Eugene, OR, USA) for 40 min and then counter stained with propidium iodide (PI; 1:6000, Sigma Aldrich, Saint Louis, MO, USA) for 30 s.

Free-floating sections (40 µm) from the Bregma, −2.3 to −6.3 mm, were used for BrdU and DCX staining. Nine sections per brain were chosen from every 8th section throughout the full-length of the DG. Cell survival was examined using a BrdU marker (Sigma Aldrich, Saint Louis, MO, USA) that was modified from a prior study [39,40]. Sections were incubated with monoclonal anti-BrdU antibody (1:100, Abcam, Cambridge, UK) in blocking solution at 4 °C overnight. The next day, sections were incubated with antibody Alexa Fluor 568 (1:300, Invitrogen, Carlsbad, CA, USA). Lastly, the sections were counter stained with diamidinophenylindole (DAPI, 1:6000, Molecular probes, Eugene, OR, USA) for 30 s at room temperature. DCX staining was used to quantify immature neurons. Sections were incubated with goat anti-DCX antibody (1:100, Santa Cruz, Dallas, TX, USA) in blocking solution overnight at 4 °C. After washing with 0.3%TritionX-100 in the PBS, the sections were incubated with antibody Alexa Fluor 488 (1:500, Invitrogen, Carlsbad, CA, USA) for 1 h in a dark chamber. Finally, sections were counter stained with PI (1:6000, Sigma Aldrich, Saint Louis, MO, USA) and cover-slipped.

### 2.5. Microscope Quantification

Cells stained with Ki-67, BrdU and DCX were counted within a 3-cell-body distance in the SGZ, including upper and lower blades [41] using 40× objective of fluorescence microscope (Nikon Eclipse 80i; Melville, NY, USA). The total numbers of cells stained with Ki-67-, BrdU-, and DCX-positive cells were determined by summation of the cell count from 9 sections per animal. Then, Ki-67 was multiplied by 15, whereas BrdU and DCX were multiplied by 8 [37,39,40,42].

### 2.6. Statistical Analysis

All statistical analysis was conducted using GraphPad Prism (Version 5.0; GraphPad Software Inc., San Diego, CA, USA) and SPSS (Version 19.0; SPSS Inc., Chicago, IL, USA). The data was expressed as mean ± standard error of mean (SEM). Student’s *t*-test and one-way ANOVA were carried out to evaluate data. *p* < 0.05 was examined to show statistical significance. 

## 3. Results

### 3.1. Hsd Ameliorated the Behavioral Deficits Caused by MTX

#### 3.1.1. Novel Object Location (NOL) Test

**Figure 1:**
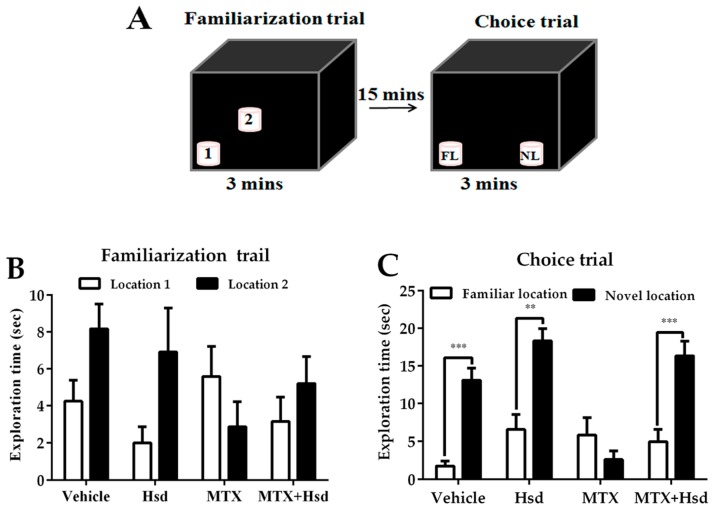
Schematic representation of the novel object location test (**A**). Mean exploration times (mean ± SEM) in the familiarization (**B**) and choice (**C**) trials of the novel object location test. All rats spent similar amounts of time exploring the objects placed in both locations 1 and 2 (*p* > 0.05) in the familiarization trial. In the choice trial, the rats in the vehicle, Hesperidin (Hsd), and Methotrexate (MTX) + Hsd groups spent a significantly longer period of time investigating the object in the novel location when compared to the object in the familiar location (** *p* < 0.01, *** *p* < 0.001), but the rats receiving MTX alone did not (*p* > 0.05). FL: Familiar location; NL: New location.

**Figure 2:**
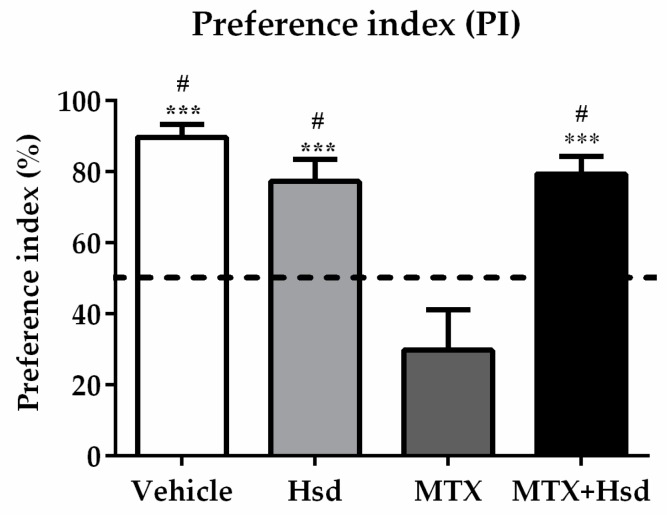
Significant differences were detected among the four groups in terms of preference index (PI) in the novel object location test. The PIs of the vehicle, Hsd, and MTX + Hsd groups were significantly different from 50% chance (# *p* < 0.05), but that of the MTX group was lower than 50%. The dashed line indicates 50% chance. The PI measured in the MTX group differed significantly from the vehicle, Hsd, and MTX + Hsd groups (*** *p* < 0.001).

In the familiarization trial (Figure 1A), the investigation time of the two identical objects was similar in all groups (*p* > 0.05, paired sample *t*-test, Figure 1B). In the choice trial (Figure 1A), the vehicle, Hsd, and Hsd + MTX groups used a significantly longer time to examine the object placed in the novel location compared to that in the familiar location (vehicle group: *** *p* < 0.001, Hsd group: ** *p* < 0.01, and Hsd + MTX group: *** *p* < 0.001, paired sample *t*-test, Figure 1C). By contrast, the MTX group spent an equal time investigating the two objects (*p* > 0.05), indicating memory impairment after receiving MTX. In addition, significant differences were found in terms of PI among the groups (* *p* < 0.05, one-way ANOVA). The PIs in the vehicle, Hsd, and MTX + Hsd groups were significantly greater than 50% chance (mean ± SEM; vehicle group: 80.12 ± 7.58, Hsd group: 73.07 ± 5.20, MTX + Hsd group: 73 ± 8.71, # *p* < 0.05, one sample *t*-test, Figure 2), indicating normal memory ability. By contrast, the PI measured in the MTX group was less than 50% chance (mean ± SEM; 10.58 ± 5.47, *p* > 0.05). Further Bonferroni post-hoc analysis showed that the PI measured in the MTX group differed significantly from the vehicle, Hsd, and MTX + Hsd groups (vehicle group: *** *p* < 0.001, Hsd group: *** *p* < 0.001, MTX + Hsd group: *** *p* < 0.001, Figure 2). These data indicate that Hsd prevented the memory deficit caused by MTX. In addition, no significant differences were detected among the groups in terms of mean velocity and total distance moved (*p* > 0.05, Table 1), indicating that MTX and Hsd administration had no effect on movement.

#### 3.1.2. Novel Object Recognition (NOR) Test

**Figure 3:**
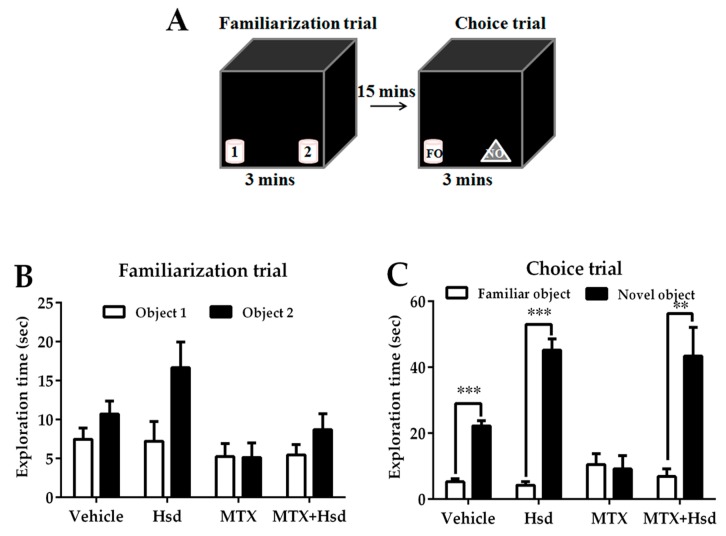
Schematic representation of the novel object recognition test (**A**). Mean exploratory times (mean ± SEM) in the familiarization (**B**) and choice (**C**) tests of the novel object recognition test after treatment. In the familiarization test, all animals spent a similar amount of time exploring objects 1 and 2 (*p* > 0.05). In the choice trail, the animals in the vehicle, Hsd, and MTX + Hsd groups spent a significantly longer period of time investigating the novel object when compared to the familiar object (** *p* < 0.01, *** *p* < 0.001), but the animals in the MTX group did not (*p* > 0.05). FO: Familiar object; NO: Novel object.

**Figure 4:**
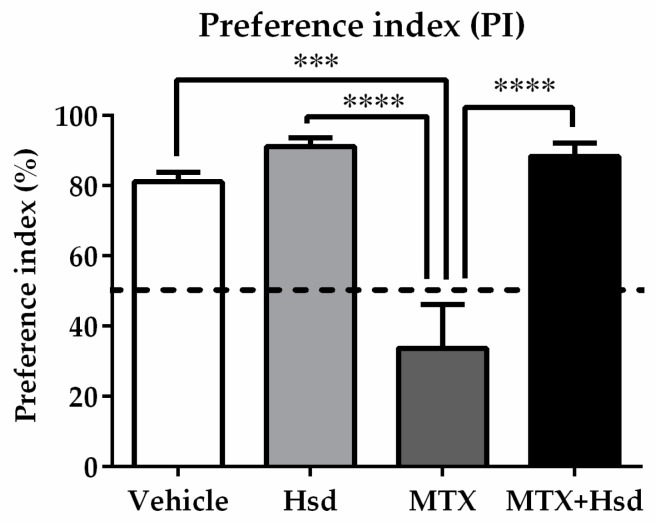
Significant differences among the four groups were found in terms of preference index (PI) in the NOR test. The PI measured in the MTX group differed significantly from those found the other three groups (*** *p* < 0.001, **** *p* < 0.0001). The PIs measured in the vehicle, Hsd, and MTX+Hsd groups were significantly higher than 50% chance (# *p* < 0.05), however that of the MTX group was lower than 50%. The dashed line indicates 50% chance.

In the familiarization trial (Figure 3A), animals from four groups spent equivalent time investigating the novel and familiar objects (*p* > 0.05, paired sample *t*-test, Figure 3B). During the choice trial (Figure 3A), the vehicle, Hsd, and MTX+Hsd groups spent a longer time investigating the novel object than the familiar object (vehicle group: *** *p* < 0.001, Hsd group: *** *p* < 0.001, and MTX + Hsd group: ** *p* < 0.01, respectively; paired Student *t*-test, Figure 3C). By contrast, rats in the MTX group showed a similar preference for both objects (*p* > 0.05), indicating that MTX induced memory impairment. Analysis of variance demonstrated a significant difference in terms of PI measured in the four groups (* *p* < 0.05). The PIs of the vehicle, Hsd, and MTX + Hsd groups were significantly higher than 50% chance (mean ± SEM; vehicle group: 77.80 ± 2.97, Hsd group: 88.51 ± 2.52, MTX + Hsd group: 82.86 ± 3.763, # *p* < 0.05, one sample *t*-test, Figure 4), suggesting that the animals had intact memory and preferred the novel object to the familiar object. Animals in the MTX group exhibited memory impairment in that their PI was not distinguishable significantly from 50% chance (mean ± SEM; 28.79 ± 15.41, *p* > 0.05). A Bonferroni post-hoc analysis confirmed that the PIs measured in the vehicle, Hsd, and MTX + Hsd groups differed significantly from the MTX group (vehicle group: *** *p* < 0.001, Hsd group: **** *p* < 0.0001, MTX + Hsd group: **** *p* < 0.0001, Figure 4). This data suggests that Hsd enhances the memory ability of MTX-treated animals. No significant differences in terms of mean velocity and total distance moved were found among the four groups after treatment (*p* > 0.05, Table 1). These effects show that MTX and Hsd administration did not have a negative effect on locomotor activity in the adult rats in the present study. 

### 3.2. Cell Proliferation, Survival and Immature Neurons after Drug Administration

#### 3.2.1. Effects of Hsd and MTX on Cell Proliferation

**Figure 5:**
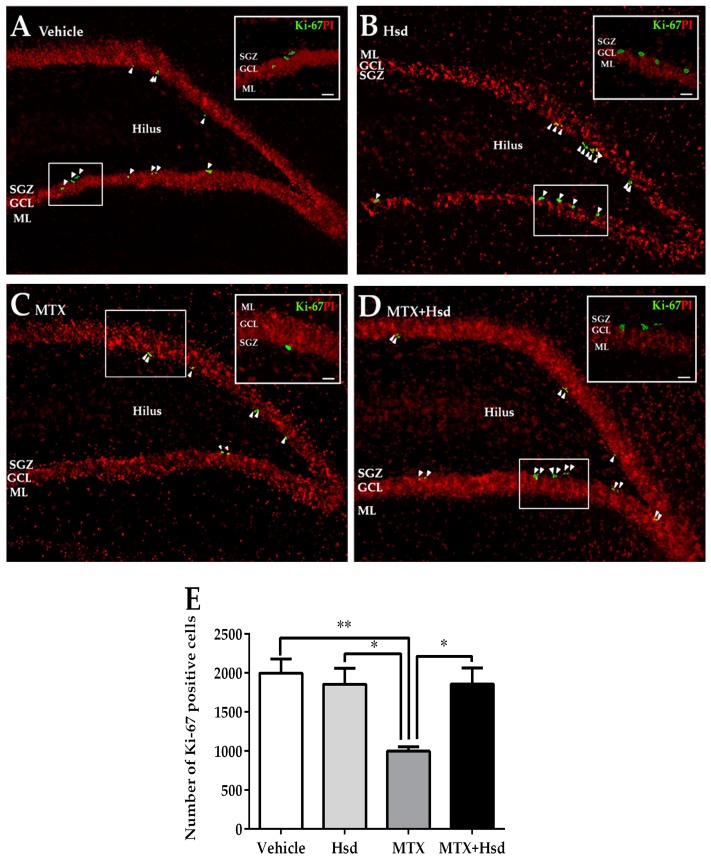
Cells stained with Ki-67 (green) in the SGZ of the DG in the hippocampus in the vehicle group (**A**), Hsd group (**B**), MTX group (**C**), and MTX + Hsd group (**D**). The results are represented as mean ± SEM. The vehicle, Hsd, and MTX + Hsd groups showed significantly higher cells stained with Ki-67 than the MTX group (Data are displayed as mean ± S.E.M., * *p* < 0.05; ** *p* < 0.01 significantly different from the MTX group) (**E**). SGZ: subgranular zone; GCL: granule cell layer; ML: molecular layer.

Ki-67 staining was performed to examine the number of newborn cells in the SGZ of the DG in the hippocampus (Figure 5). The mean numbers of cells stained with Ki-67 were 1998 ± 180.3, 1855 ± 205.0, 1000 ± 53.34, and 1858 ± 203.0 in the vehicle, Hsd, MTX, and MTX+Hsd groups, respectively. One-way ANOVA demonstrated that the number of cells stained with Ki-67 significantly differed among the groups (** *p* < 0.01). A Bonferroni post-hoc test proved that animals receiving MTX alone experienced a significant decrease in cells stained with Ki-67 compared to the vehicle group (** *p* < 0.01, Figure 5E). By contrast, the numbers of cells stained with Ki-67 in the Hsd and MTX + Hsd groups did not significantly vary from the vehicle group (*p* > 0.05, Figure 5E). However, the numbers of cells stained with Ki-67 in the Hsd and Hsd + MTX groups were significantly higher than that of the MTX group (* *p* < 0.05, Figure 5E). These outcomes reveal that preventive action of Hsd treatment can ameliorate the decreases in cell proliferation caused by MTX treatment.

#### 3.2.2. Effects of Hsd and MTX on Cell Survival

**Figure 6:**
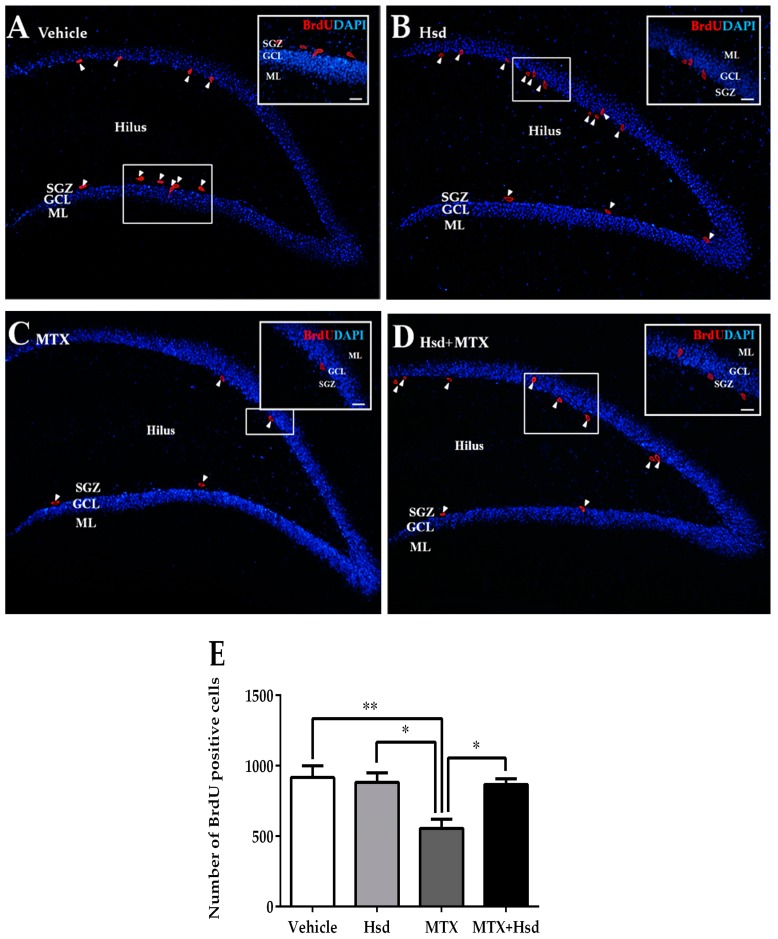
BrdU positive cells (red) in the SGZ of the DG in the hippocampus in the vehicle group (**A**), Hsd group (**B**), MTX group (**C**), and MTX + Hsd (**D**) group. The data shows a significant decline in cell survival in the MTX group in comparison with the vehicle, Hsd, and MTX + Hsd groups (Data are displayed as mean ± S.E.M. * *p* < 0.05; ** *p* < 0.01 significantly different from the MTX group) (**E**). SGZ: subgranular zone; GCL: granule cell layer; ML: molecular layer.

Cell survival in the SGZ of the DG in the hippocampus was measured using BrdU (Figure 6). The mean numbers of BrdU cell count per animal were 917 ± 83.74, 883 ± 68.53, 556 ± 63.22, and 868 ± 40.63 in the vehicle, Hsd, MTX, and MTX + Hsd groups, respectively. The number of BrdU cell count was significantly inconsistent among the four groups (** *p* < 0.01, one-way ANOVA, Bonferroni post hoc test). That of the MTX group was significantly lower than that of the vehicle group (** *p* < 0.01, Bonferroni post hoc test, Figure 6E). However, those of the Hsd and MTX + Hsd groups were not significantly consistent with that of the vehicle group (*p* > 0.05, Figure 6E), but were significantly higher than that of the MTX group (* *p* < 0.05, Figure 6E). The results suggest that receiving MTX decreased viability of cells in the SGZ of the DG in the hippocampus, which was improved by co-administration with Hsd.

#### 3.2.3. Effects of Hsd and MTX on Immature Neurons

**Figure 7:**
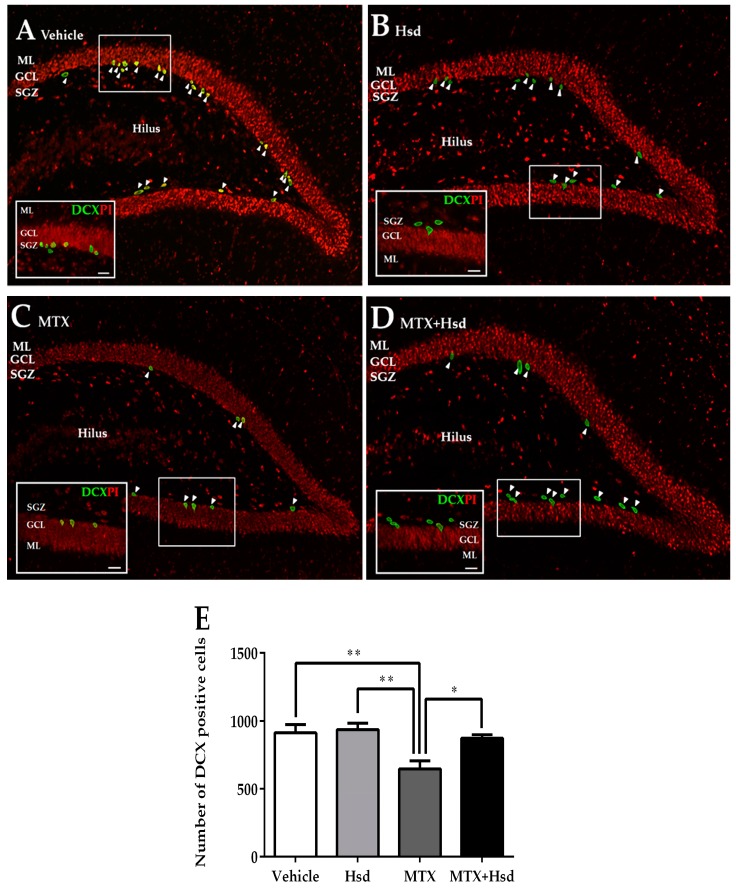
Immunofluorescence staining for DCX (green) in the SGZ of the DG in the hippocampus in the vehicle group (**A**), Hsd group (**B**), MTX group (**C**), and MTX + Hsd (**D**) group. The animals in the vehicle, Hsd, and MTX + Hsd groups exhibited more cells stained with DCX than the MTX group (Data represent mean ± S.E.M, * *p* < 0.05 and ** *p* < 0.01, differed significantly from the MTX group) (**E**). SGZ: subgranular zone; GCL: granule cell layer; ML: molecular layer.

Levels of immature neurons in the SGZ of the DG in the hippocampus were quantified using DCX positive cells (Figure 7). The mean numbers of cells stained with DCX were 914.7 ± 61.05, 938.7 ± 47.40, 648.0 ± 58.46, and 873.3 ± 26.89 in the vehicle, Hsd, MTX, and MTX + Hsd group, respectively. The numbers of DCX positive cells differed significantly when compared by all of the four groups of rats (** *p* < 0.01, one-way ANOVA, Bonferroni post hoc test). A significant decrease in cells stained with DCX was revealed in animals receiving MTX alone when compared with rats in the vehicle group (** *p* < 0.01, Bonferroni post hoc test, Figure 7E), whereas animals in the Hsd and Hsd + MTX groups did not differ from the animals in the vehicle group (*p* > 0.05). Moreover, the numbers of cells stained with DCX in the Hsd and Hsd + MTX groups were significantly higher than the MTX group (** *p* < 0.01 and * *p* < 0.05, respectively, Figure 7E). These outcomes show that co-administration of Hsd with MTX can increase the number of cells stained with DCX in the SGZ of the DG in the hippocampus.

## 4. Discussion

The present study demonstrates the neuroprotective activities of Hsd on the memory deficits associated with reductions in hippocampal neurogenesis after MTX treatment. The results reveal that animals treated with MTX developed memory deficits and exhibited decreased neurogenesis. However, co-administration of Hsd alleviated impairments to memory and hippocampal neurogenesis.

In the present study, both the NOL and NOR tests were used to observe the actions of Hsd and MTX on cognition in animals. Neither the NOL nor the NOR test requires training, external motivation, or reinforcements. These tasks require an intact hippocampus for encoding, consolidation, and retrieval, which are cognitive processing including learning and memory [43,44]. These tests assess the behavior of rats when they are exposed to locations or objects. It relies on rats’ natural preference for exploring novelty; if they recognize objects or locations of objects from their previous exposure, they will use more time observing novel objects or objects in new locations [45]. Rats with hippocampal lesions have impaired memory and, consequently, demonstrate no favor for novel locations or objects [46].

In our study, the MTX group demonstrated memory impairment in both the NOL and NOR tests. In the NOL test, MTX rats did not like the object in the novel location, indicating MTX-induced memory deficit. This is in agreement with the conclusions of Lyons et al. (2011), who have investigated the side effects of MTX (75 mg/kg) on spatial memory using NOL and Morris water maze (MWM) tests [6]. Animals in the MTX group in our study also expressed no preference for any of the objects in the NOR test, indicating memory impairment. These outcomes are compatible with earlier studies, which also found that receiving MTX produces cognitive deficit as measured by the NOR test [17,47]. By contrast, animals receiving co-treatment with MTX and Hsd did not exhibit memory impairment in either the NOL or the NOR test. Thus, these results reveal that Hsd can ameliorate the memory deficits caused by MTX treatment. Previous studies have also found that administration of Hsd can increase memory ability [34,48,49,50]. One study demonstrated that the administration of Hsd at a dosage of 100 mg/kg for 21 days significantly restores memory impairment in rat models of Alzheimer’s disease (AD) [49]. The potential anti-oxidative and anti-inflammatory effects of Hsd may be mechanisms by which Hsd improves cognitive function [49].

In addition, various studies have demonstrated that spatial memory is actively correlated with changes of hippocampal neurogenesis [37,51,52]. Injury of neurogenesis is a possible action by which MTX induces cognitive deficit [17,20]. We, thus, examined the effects of Hsd and MTX on proliferating cells (Ki-67 positive cell expression), cell survival (BrdU positive cell expression), and immature neurons (DCX positive cell expression) in the hippocampal DG, which have important functions in the memory action. Levels of the protein, Ki-67, increase during the process of cell division in all phases of the active cell cycle, but it is not found in the G0 phase [53,54]. An exogenous thymidine analog, BrdU, is incorporated into DNA in the cell during the S-phase of the cell cycle. It is still leftover in the nucleus after finishing mitosis [55]. In the present investigation, the numbers of cells stained Ki-67 and BrdU were reduced in MTX-treated animals, indicating a decline in cell proliferation and survival. These results confirm previous reports that found that MTX treatment reduces cell proliferation and survival in the SGZ of the DG [6], which results in memory impairment during MTX treatment. Recently, it has been reported that MTX is capable of passing the blood–brain barrier (BBB), which leads to neurotoxicity [15,56,57]. It can disturb the synthesis of pyrimidine and purine, which are required for DNA and RNA synthesis. This consequently inhibits cell proliferation and induces apoptosis [13,14]. In addition to decreasing neurogenesis in the hippocampus, MTX also disrupts memory processes [20]. This suggests that the memory impairments we observed in the MTX group may be due to the disruption of cell proliferation and survival in the hippocampus.

However, the co-treatment of Hsd with MTX increased the quantity of Ki-67 and BrdU positive cells to the control levels after 21 days of treatment. Previous examinations have displayed that Hsd increases antioxidant activities [49], which can reduce reactive oxygen species (ROS) and mitochondrial dysfunction [58]. Since Hsd has the ability to cross the BBB, it may further have inhibitory effects on neuro-inflammation [59]. These effects may protect against neuronal damage. One of the adverse effects of MTX treatment is that it causes increases in reactive oxygen species (ROS) in the central nervous system (CNS, which may reduce antioxidant activity and lead to neuronal toxicity, neuro-inflammation, and neuronal cell death [21]. Therefore, adding Hsd to MTX treatment could restore cell proliferation and survival by improving antioxidant pathways.

In addition to cell proliferation and survival, we also measured the number of immature neurons accessed by DCX positive cells. A microtubule-associated protein, DCX, expresses in adult-born cells to immature neurons. It is generally used as a marker indicating changes of neurogenesis [60]. In the present analysis, the number of DCX positive cells was suppressed in the MTX group, showing that MTX treatment reduced the numbers of immature neurons. Similarly, previous studies have discovered that MTX reduces the number of both Ki-67- and DCX-positive cells in the adult hippocampus [6,15]. However, administration of Hsd attenuated the reduction of immature neurons caused by MTX treatment. This data supports the neuroprotective activity of Hsd previously described in rats, that it enhances levels of brain-derived neurotrophic factor (BDNF) in the hippocampus [61], is involved in synaptic plasticity, and improves learning ability and memory functions [62,63]. Hsd also modulates the multiple cellular signaling pathways such as phosphatidylinositol-3-kinase (PI3K), mitogen-activated protein kinase (MAPK), and protein kinase C (PKC), which are important in the control of cellular functions [64].

## 5. Conclusions

In summary, the present study confirms that MTX is able to decrease hippocampal neurogenesis and leads to memory impairment in adult rats. However, Hsd significantly ameliorated the memory impairment in MTX-treated rats by restoring levels of neurogenesis in the DG of the hippocampus. Further investigation to measure the BDNF levels and antioxidant activities within the hippocampus may assist to clarify the actions of Hsd, which acts on memory and levels of neurogenesis caused by MTX treatment.

## Figures and Tables

**Table 1:** Mean velocity and total distance moved of the animals in the novel object location (NOL) and novel object recognition (NOR) tests after treatment.

	Novel Object Location (NOL)		Novel Object Recognition (NOR)	
	Mean Velocity (cm/s)	Total Distance Moved (cm)	Mean Velocity (cm/s)	Total Distance Moved (cm)
Control	0.91 ± 0.14	9620.33 ± 2357.44	0.94 ± 0.29	2159.59 ± 683.9
Hsd	1.09 ± 0.74	9696.423 ± 2983.63	0.99 ± 0.23	2104.29 ± 123.3
MTX	1.13± 0.37	10585.58 ± 1290.11	0.78 ± 0.23	2484.98 ± 265.8
MTX+Hsd	1.19 ± 0.27	10904.55 ± 3819.26	0.87 ± 0.48	2524.63 ± 285.4

Values are represented as mean ± SEM.
